# Cytolytic Activity of Effector T-lymphocytes Against Hepatocellular Carcinoma is Improved by Dendritic Cells Pulsed with Pooled Tumor Antigens

**DOI:** 10.1038/s41598-019-54087-0

**Published:** 2019-11-27

**Authors:** Thaweesak Chieochansin, Chutamas Thepmalee, Janya Grainok, Mutita Junking, Pa-thai Yenchitsomanus

**Affiliations:** 1 Siriraj Center of Research Excellence for Cancer Immunotherapy, Faculty of Medicine Siriraj Hospital, Mahidol University, Bangkok, Thailand; 2 Graduate Program in Immunology, Department of Immunology, Faculty of Medicine Siriraj Hospital, Mahidol University, Bangkok, Thailand; 3 Division of Biochemistry, School of Medical Science, University of Phayao, Phayao, Thailand; 4 International Graduate Program in Medical Biochemistry and Molecular Biology, Department of Biochemistry, Faculty of Medicine Siriraj Hospital, Mahidol University, Bangkok, Thailand

**Keywords:** Cancer therapy, Gastrointestinal cancer, Tumour immunology

## Abstract

Cellular immunotherapy is a promising new therapeutic approach for hepatocellular carcinoma (HCC), which has a high recurrence rate, irrespective of the treatment administered. In this study, we attempted to improve the cytolytic activity of effector T-lymphocytes against HCC. T-lymphocytes were activated by monocyte-derived dendritic cells (DCs) pulsed with cell lysate or RNA prepared from HCC cell lines. Monocytes were activated for differentiation into DCs by treatment with the IL4 and GM-CSF. DCs were pulsed with cell lysate or RNA prepared from a single cell line or combinations of two or three HCC cell lines, and then co-cultured with autologous T-lymphocytes with the intent of creating specific cytotoxicity. We discovered that DCs pulsed with total RNA effectuated greater T-lymphocyte function than DCs pulsed with total cell lysate, as evidenced by greater cytolytic activities against HCC target cells. The percentage of Huh7, HepG2, and SNU449 cell apoptosis at effector:target ratio of 10:1 was 42.6 ± 4.5% (*p* = 0.01), 33.6 ± 3.1% (*p* = 0.007), and 21.4 ± 1.4% (*p* < 0.001), respectively. DCs pulsed with pools of antigens prepared from three cell lines improved the cytolytic function of effector T-lymphocytes by approximately two-fold (*p* < 0.001), which suggests that this approach be further developed and applied for adoptive transfer treatment of HCC.

## Introduction

Hepatocellular carcinoma (HCC) is a major public health problem worldwide, and it is the fifth most common cancer^1^. The incidence of HCC is higher in Asia than in America and Europe^2^. In Thailand, it is the leading cause of cancer death in males, and the second most common cancer among females^3^. Standard treatments for HCC include surgery, radiofrequency ablation (RFA), microwave ablation, percutaneous ethanol injection (PEI), transarterial chemoembolization (TACE), radioembolization, cryoablation, radiation therapy, stereotactic radiotherapy, systemic chemotherapy, and liver transplantation^4^. However, a high recurrence rate is generally reported in these patients, irrespective of the treatment administered^5^. Targeted therapy using multikinase inhibitors (Serafernib^6^ or Regorafenib^7^) and immunotherapy using antibody blocking programmed cell death receptor-1 (PD-1) (Nivolumab^8^) have been recently been approved as second-line drug treatments for HCC, and this has raised hopes of improved survival. However, the survival rates of HCC patients after treatment with these novel drugs are still low^6–9^. Thus, alternative strategies for treatment of HCC are urgently required.

Cellular immunotherapy is a promising new therapeutic approach for cancer. Immunotherapy involves the uses of a patient’s own immune cells, which are activated or engineered and then proliferated *ex vivo* for transfer back into the patient to attack and kill cancer cells. Dendritic cell (DC) based vaccines have shown some benefits in the treatment of HCC^10–12^, and this has resulted in the initiation of clinical trials for other types of cancer^13–16^. However no satisfactory response or stable disease outcome has yet been reported from the majority of early-phase clinical trials^17,18^.

The failure of DC-based immunotherapy in patients with advanced stage cancer might be explained by DC dysfunction or the presence of immunosuppressive cells and cytokines generated during the course of disease from cancerous and non-cancerous cells that inhibit T-lymphocyte activation^19,20^. It has, therefore, been proposed that the use of activated T-lymphocytes instead of DCs may overcome these obstacles^21^. The effector T- lymphocytes used in this approach are cytotoxic T- lymphocytes and chimeric antigen receptor (CAR) T-lymphocytes^22,23^. Cytotoxic T-lymphocytes can be activated by DCs pulsed with tumor associated antigens (TAAs) that are processed via proteasome to present as specific peptide antigens on major histocompatibility complex (MHC) to activate T-lymphocyte receptors (TCRs)^24,25^. Activated effector T-lymphocytes are then transferred into the patient to combat cancer cells^24,25^. Several solid cancers, including melanoma^26^, renal cancer^27^, colorectal cancer^15,28^, and cholangiocarcinoma (CCA)^29^, that contain TTAs have been employed for DC-activation of T-lymphocytes to kill cancer cells.

However, there are several unmet needs in this experimental setting. Firstly, TAAs used to pulse DCs may have a limitation of MHC restriction^30^. Secondly, a high diversity of cancer cell population within tumor mass, which is referred to as intra-tumor heterogeneity, was reported in several tumors^31,32^, and this results in varied antigen expression within the same tumor mass^33^. Thirdly, the mixture of cancer cell sub-population within individual HCC patients might also be a problem, since this can lead to therapeutic resistance and increased recurrence rate^34,35^. Although total cell lysate or total RNA from tumor mass or pools of cancer cell lines could boost the extent of multiple-epitope antigens for pulsing DCs, the data from the reported studies were equivocal^36–39^. Our previous study in cholangiocarcinoma (CCA) revealed that T-lymphocytes activated with DCs pulsed with total RNAs had higher killing ability to CCA cells than that activated with DCs pulsed with cell lysate. In addition, T-lymphocytes activated with DCs pulsed with pooled mRNAs from more than one cell line showed greater cytolytic activities than those activated with DCs pulsed with mRNAs from a single cell line^29^. Consistent with that finding, we hypothesized for this study that the cytolytic activity of T-lymphocytes activated with DCs pulsed with pooled TAAs prepared from multiple HCC cell lines would yield greater specific cytolytic activity. We tested this hypothesis by determining the cytolytic activities of effector T-lymphocytes activated with DCs pulsed with pooled RNAs and cell lysates from multiple HCC cell lines to compare their efficacies. Our investigation revealed significantly improved cytolytic activity of effector T-lymphocytes against HCC cell lines.

## Results

### Generation of monocyte-derived dendritic cells

Monocytes are adhesive cells that bind to culture plate. The advantage of this property was taken to use for isolation of monocytes out of other peripheral blood mononuclear cells (PBMCs). Monocytes were isolated from PBMCs prepared from blood samples of 5 healthy volunteers. Then, the isolated monocytes were differentiated into immature dendritic cells (iDCs) by cultivation in AIM-V medium supplemented with GM-CSF and IL4 for 5 days. After that, iDCs were pulsed with total RNAs or total cell lysates prepared from single, combination of two or three HCC cell lines and cultured in AIM-V medium supplemented with TNFα and IFNγ, in which iDCs were further differentiated into mature dendritic cells (mDCs). Phenotypic markers, including monocyte marker (CD14), DC marker (CD11c), DC maturation marker (CD83), T-cell co-stimulatory markers (CD40 and CD86), and MHC class II (HLA-DR), were investigated by flow cytometry.

**Figure 1:**
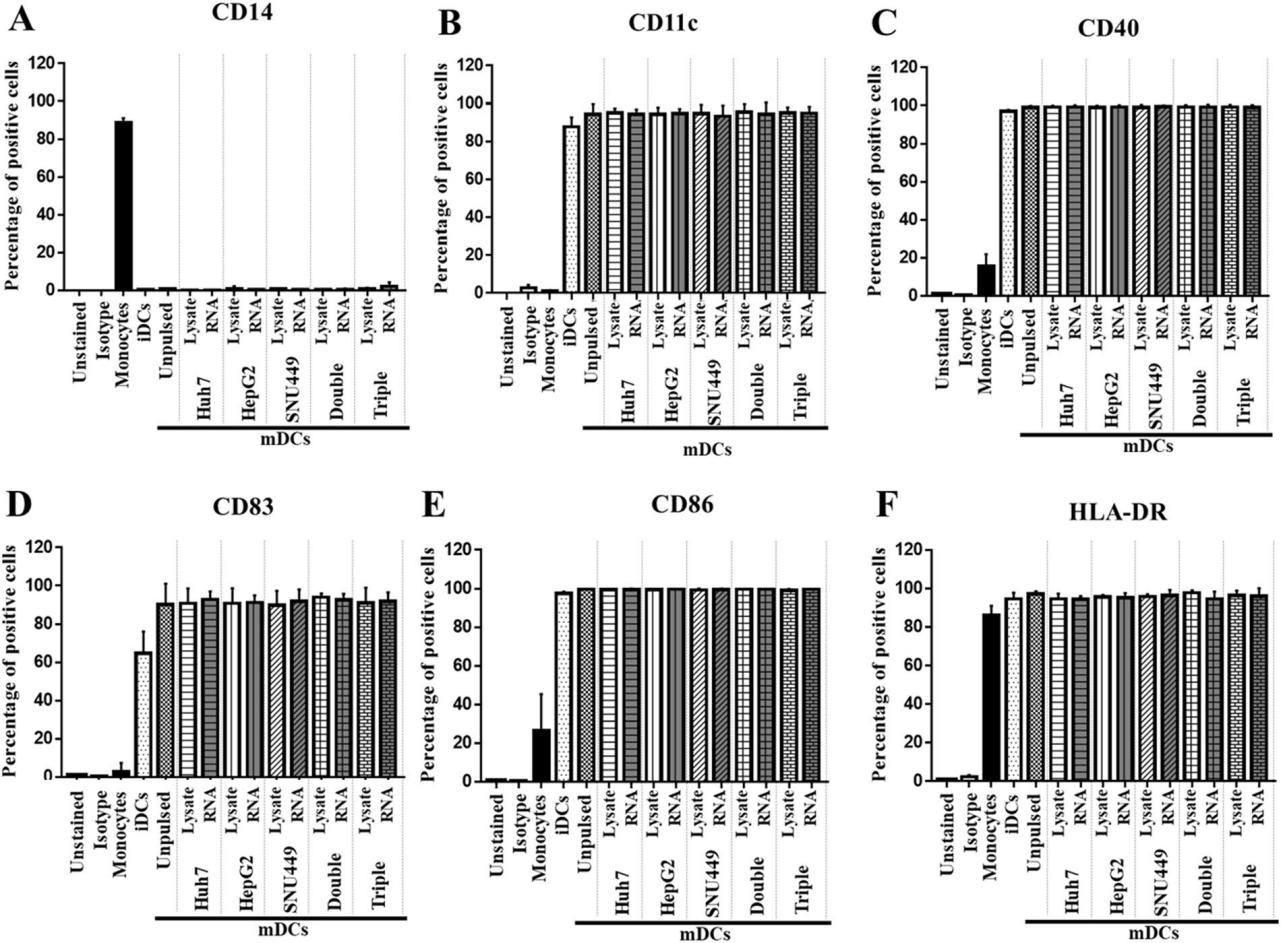
Expression levels of monocyte markers, maturation markers, and co-stimulatory molecules on monocytes, iDCs, and mDCs. Monocytes were retrieved from adherent PBMCs. iDCs were induced from monocytes by supplementation with GM-CSF and IL4 for 6 days. mDCs were further driven from iDCs with TNFa and IFNg for 2 days. During their maturation, DCs were pulsed with total cell lysate or RNA prepared from single, double, or triple HCC cell lines. Monocytes, iDCs, and mDCs were stained with indicated antibodies and then evaluated for fluorescent signals by flow cytometry. The plotted bar graph indicates percentages of positive cells (mean ± SEM) that exhibited monocyte markers, CD14 (**A**); DC markers, CD11c (**B**); T-cell costimulatory molecules, CD40 (**C**), and CD86 (**E**); and, maturation markers, CD83 (**D**) and HLA-DR (**F**).

Monocyte marker (CD14) was found in only monocyte state (88.7% ± 2.4%), and it disappeared when the cells were driven as iDCs and mDCs (Fig. 1A). In contrast, the expression levels of CD11c were highly increased when the cells were differentiated as iDCs (87.7% ± 1.5%) and mDCs (94.3% ± 5.4%) (Fig. 1B). The levels of co-stimulatory molecules and maturation markers, including CD40 and CD83, CD86, and HLA-DR, were also increased in both iDCs (CD40: 96.9 ± 0.8%, CD83: 64.8 ± 11.4%, CD86: 97.5 ± 1.0%, and HLA-DR: 94.6 ± 3.2%) and mDCs (CD40: 99.0 ± 0.9%, CD83: 90.2 ± 0.1%, CD86: 99.8 ± 0.1%, and HLA-DR: 97.2 ± 1.4%) when compared with monocyte state (CD40: 15.8 ± 6.2%, CD83: 3.0 ± 4.4%, CD86: 26.4 ± 19.0%, and HLA-DR: 86.1 ± 4.9%) (Fig. 1C–F). The expression levels of these markers were not significantly different when different sources of antigens were used to pulse mDCs (Fig. 1B–F). Cell morphologies of monocytes, iDCs, and mDCs under light microscope are presented in Supplementary Fig. S1. Using this experimental protocol, monocytes were successfully differentiated as iDCs and then as mDCs.

### Effector T-lymphocyte population after activation with mDCs

**Figure 2:**
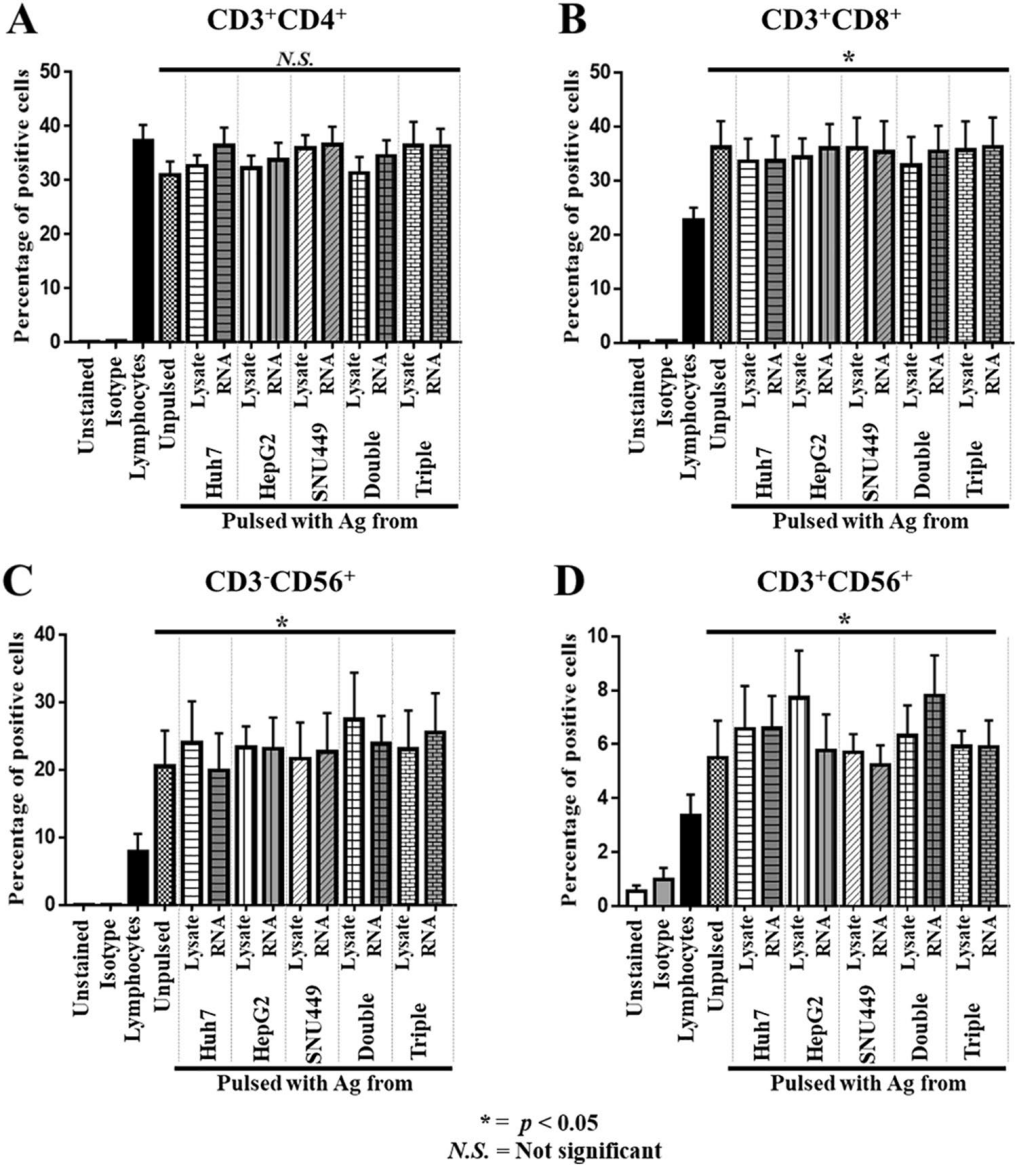
Expression of lymphocyte markers on effector T-lymphocytes after activation with DCs pulsed with antigens. Autologous lymphocytes (non-adhering PBMCs) were activated to become effector T-lymphocytes by co-culturing with DCs pulsed with cell lysate or RNA prepared from single, double, or triple HCC cell lines for 2 days. Effector T-lymphocytes were then further propagated in AIM-V medium supplemented with IL2, IL7, and IL15 for 10 days. The expressions of CD3+, CD4+, CD8+, and CD56+ were examined by flow cytometry after staining with specified antibodies. The percentages (mean ± SEM) of specific populations of effector cells, including CD3+ CD4+ helper T-cells (**A**), CD3+ CD8+ cytotoxic T-cells (**B**), CD3-CD56+ NK cells (**C**), and CD3+ CD56+ NKT cells (**D**) were calculated from three independent experiments. (*p < 0.05 compared with non-adhering PBMCs. Data analyzed by one-way ANOVA with Tukey’s correction).

mDCs were used to activate autologous T-lymphocytes by co-culturing at a mDC:lymphocyte ratio of 1:10 for 2 days. After 10 days of activation, phenotypic markers of T-lymphocytes (CD3), helper T-lymphocytes (CD4), cytotoxic T-lymphocytes (CTLs) (CD8), and NK cells (CD56) were analyzed by flow cytometry. The percentages of T-helper lymphocytes (CD3^+^CD4^+^) after activation (31.0 ± 6.0%) were not significantly different from those without activation (37.2 ± 7.3%) (Fig. 2A). However, CTLs (CD3^+^CD8^+^) after activation were significantly increased (36.1 ± 12.0%) when compared with those without activation (22.6 ± 5.7%) (Fig. 2B). NK cells (CD3^−^CD56^+^) and NKT (CD3^+^CD56^+^) were significantly increased (20.5 ± 8.9% for NK, and 5.5 ± 3.3% for NKT) when compared with those without activation (7.8 ± 2.5% for NK, and 3.3 ± 2.0% for NKT) (Fig. 2C–D). The percentages of helper T-lymphocytes, CTLs, NK cells, and NKT cells were not significantly different among groups of effector T cells that were activated with mDCs pulsed with different sources of antigens (Fig. 2A–D). These data show that the number of effector cells were increased after activation with mDC pulsed with different sources of antigens.

### IFNγ production of effector T-lymphocytes

**Figure 3:**
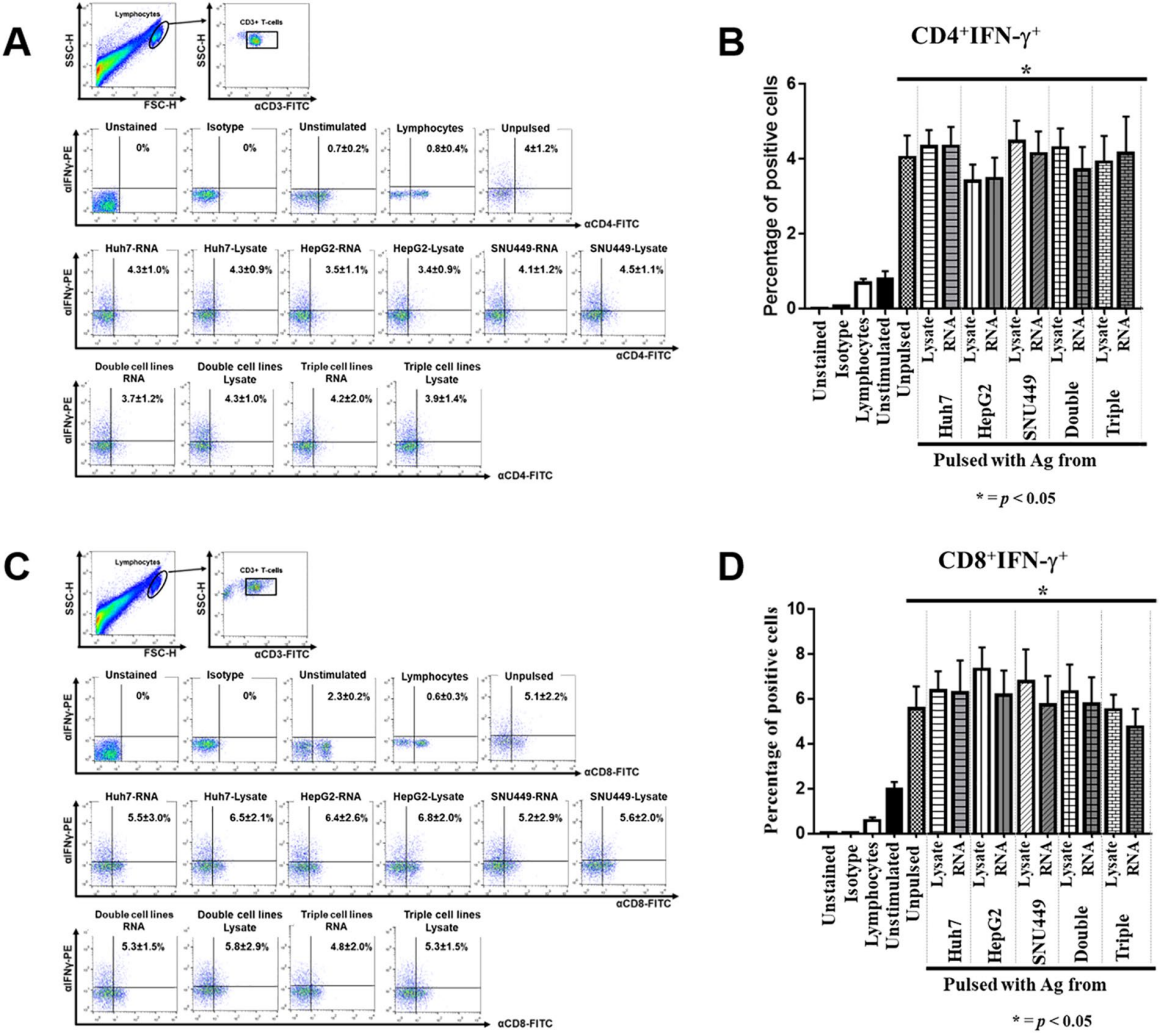
IFN-γ production profile of helper T-cells and cytotoxic T-cells in effector T-lymphocytes. After activation and propagation for 10 days, effector T-lymphocytes were treated with PMA and ionomycin for 24 hours, and then investigated for intracellular IFNγ production by flow cytometry. Effector T-cells were primarily gated by CD3+ cells, and then the IFNγ production profile of CD4 + helper T-cells (**A**) and CD8+ cytotoxic T-cells (**C**) was further analyzed. Data of CD4+ IFNγ+ (**B**) and CD8+ IFNγ+ (**D**) populations are shown as mean ± SEM, which were calculated from three independent experiments. (*p < 0.05 compared with non-adhering PBMCs. Data analyzed by one-way ANOVA with Tukey’s correction).

To investigate IFNγ of effector T-lymphocytes, the cells were treated with stimulator including PMA and ionomycin for 12 hours. Then, monensis and Beferin-A were added to inhibit the secretory pathway for following 12 hours. After performing intra-cellular IFNγ and surface marker staining, T-lymphocytes were analyzed by flow cytometry. Intracellular IFNγ production in effector T-lymphocytes was significantly increased in both CD4^+^ T-cells (4.0 ± 1.2%) and CD8^+^ T-cells (5.5 ± 2.0%) when they were individually compared with non-activated T-cell populations (0.7 ± 0.4% and 2.2 ± 0.6%, respectively) (Fig. 3A–D), which indicates that these T-lymphocytes exhibited the property of effector cells.

### Cytotoxic activities of effector T-lymphocytes activated by DCs pulsed with single source of antigen

**Figure 4:**
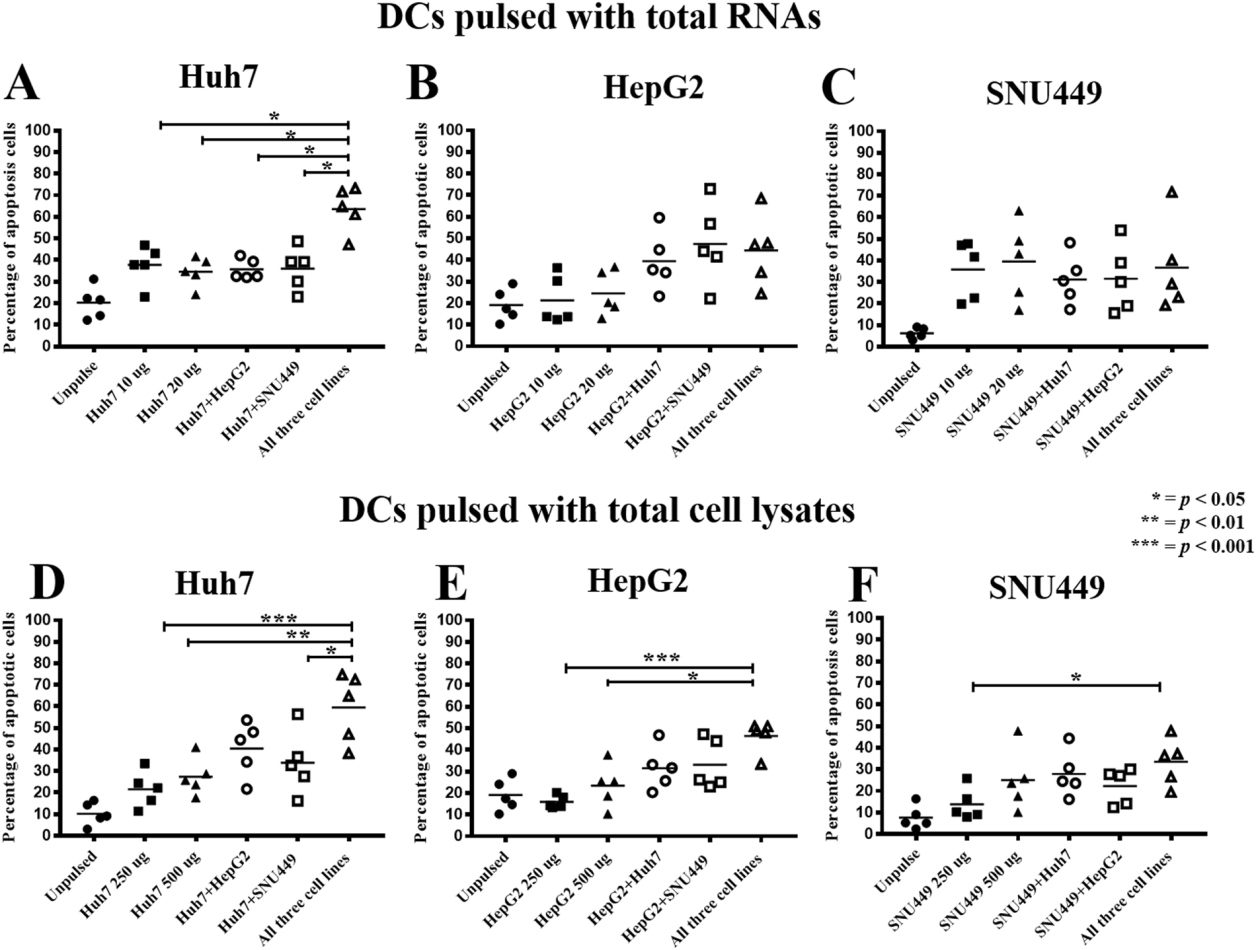
Comparison of cytotoxic activity of effector cells when activated with DCs pulsed with different sources of antigens. Data presented as scatter plots of percentages of target cell apoptosis at the most effective E:T ratio, which was 10:1. Data show percentages (mean ± SEM) of target cell apoptosis that were calculated from three independent experiments. (*p < 0.05, **p < 0.01, ***p < 0.001 analyzed by one-way ANOVA with Tukey’s correction).

Total RNA or cell lysate prepared from HCC cell lines, including Huh7, HepG2, and SNU449, was used for pulsing DCs, and then activating T-lymphocytes to become effector cells. Three different HCC cell lines (Huh7, HepG2, and SNU449) used in this study were established from patients with different backgrounds that displayed different morphologies, diverse cultivation behaviors, and distinctive protein expression profiles^40–42^ The percentages of apoptotic HCC cells in the conditions that total RNA was used to pulse DCs were significantly greater than those that were pulsed with total cell lysate for all three HCC cell lines (Fig. 4, Supplementary Fig. S2). In the condition where DCs were pulsed with RNA, the percentages of Huh7, HepG2, and SNU449 cell apoptosis at an E:T ratio of 10:1 were 42.6 ± 4.5% (*p* = 0.01), 33.6 ± 3.1% (*p* = 0.007), and 21.4 ± 1.4% (*p* < 0.001), respectively (Fig. 4, Supplementary Fig. S2). In the condition where DCs were pulsed with cell lysate, the percentages of Huh7, HepG2, and SNU449 cell apoptosis at an E:T ratios of 10:1 were 25.1 ± 5.4%, 23.1 ± 2.6%, and 11.9 ± 3.9%, respectively (Fig. 4, Supplementary Fig. S2).

### Cytotoxic activities of effector T-lymphocytes activated by DCs pulsed with combined antigens from two cell lines

The percentages of apoptotic Huh7 cells in the conditions where effector T-lymphocytes were activated by DCs pulsed with combined RNAs or cell lysates from two cell lines were higher than those in the conditions where effector T-lymphocytes were activated by DCs pulsed with antigens from single cell line, especially in the condition where DCs were pulsed with combined cell lysates (Fig. 4A,B, and Supplementary Fig. S3). In the condition where DCs were pulsed with a combination of RNAs from Huh7 and HepG2, the percentage of Huh7 cell apoptosis at an E:T ratio of 10:1 was 41.4 ± 2.0% (Fig. 4A and Supplementary Fig. S3); whereas, in the condition where DCs were pulsed with combined lysates from Huh7 and HepG2, the percentage of Huh7 cell apoptosis at an E:T ratio of 10:1 was 39.3 ± 9.3% (Fig. 4D and Supplementary Fig. S3). In the condition of where DCs were pulsed with a combination of RNAs from Huh7 and SNU449, the percentage of Huh7 cell apoptosis at an E:T ratio of 10:1 was 42.3 ± 7.0% (Fig. 4A and Supplementary Fig. S3); whereas, in the condition where DCs were pulsed with combined lysates from Huh7 and SNU449, the percentage of Huh7 cell apoptosis at an E:T ratio of 10:1 was 37.3 ± 5.1% (Fig. 4D and Supplementary Fig. S3).

In the condition where DCs were pulsed with a combination of RNAs from HepG2 and Huh7, the percentage HepG2 cell apoptosis at an E:T ratio of 10:1 was 39.1 ± 13.6% (Fig. 4B and Supplementary Fig. S3); whereas, in the condition where DCs were pulsed with pooled lysates from HepG2 and Huh7, the percentage of HepG2 cell apoptosis at an E:T ratio of 10:1 was 37.3 ± 8.4% (Fig. 4E and Supplementary Fig. S3). In the condition where DCs were pulsed with a combination of RNAs from HepG2 and SNU449, the percentage of HepG2 cell apoptosis at an E:T ratio of 10:1 was 47.4 ± 18.8% (Fig. 4B and Supplementary Fig. S3); whereas, in the condition where DCs were pulsed with pooled lysates from HepG2 and SNU449, the percentage of HepG2 cell apoptosis at an E:T ratio of 10:1 was 33.1 ± 11.5% (Fig. 4E and Supplementary Fig. S3).

In the condition where DCs were pulsed with a combination of RNAs from SNU449 and Huh7, the percentage of SNU449 cell apoptosis at an E:T ratio of 10:1 was 30.2 ± 5.4% (Fig. 4C and Supplementary Fig. S3); whereas, in the condition of where DCs were pulsed with pooled lysates from SNU449 and Huh7, the percentage of SNU449 cell apoptosis at an E:T ratio of 10:1 was 27.9 ± 10.5% (Fig. 4F and Supplementary Fig. S3). In the condition where DCs were pulsed with a combination of RNAs from SNU449 and HepG2, the percentage of SNU449 cell apoptosis at an E:T ratio of 10:1 was 29.3 ± 10.0% (Fig. 4C and Supplementary Fig. S3); whereas, in the condition where DCs were pulsed with pooled lysates from SNU449 and HepG2, the percentage of SNU449 cell apoptosis at an E:T ratio of 10:1 was 22.3 ± 8.8% (Fig. 4F and Supplementary Fig. S3).

### Cytotoxic activities of effector T-lymphocytes activated by DCs pulsed with combined antigens from three cell lines

The highest cytotoxic activities of effector T-lymphocytes against HCC cells were exhibited in the condition where DCs were pulsed with antigens prepared from a combination of three cell lines (Fig. 4, and Supplementary Fig. S4). Cytotoxic activities were improved approximately two-fold when compared with those activated by DCs pulsed with a single source of antigen (*p* < 0.001) (Fig. 4 and Supplementary Fig. S4). In the condition where DCs were pulsed with a combination of RNAs from three cell lines, the percentage of Huh7 cell apoptosis at an E:T ratio of 10:1 was 61.5 ± 13.8% (Fig. 4A and Supplementary Fig. S4); whereas, in the condition where DCs were pulsed with pooled lysates from three cell lines, the percentage of Huh7 cell apoptosis at an E:T ratio of 10:1 was 61.0 ± 19.5%%, respectively (Fig. 4D and Supplementary Fig. S4). In the condition where DCs were pulsed with a combination of RNAs from three cell lines, the percentage of HepG2 cell apoptosis at an E:T ratio of 10:1 was 44.4 ± 16.5% (Fig. 4B and Supplementary Fig. S4); whereas, in the condition where DCs were pulsed with pooled lysates from three cell lines, the percentage of HepG2 cell apoptosis at an E:T ratio of 10:1 was 49.4 ± 1.4% (Fig. 4E and Supplementary Fig. S4). In the condition where DCs were pulsed with a combination of RNAs from three cell lines, the percentage of SNU449 cell apoptosis at an E:T ratio of 10:1 was 30.8 ± 8.7% (Fig. 4C and Supplementary Fig. S4); whereas, in the condition where DCs were pulsed with pooled lysates from three cell lines, the percentage of SNU449 cell apoptosis at an E:T ratio of 10:1 was 33.4 ± 5.9% (Fig. 4F and Supplementary Fig. S4).

## Discussion

Cellular immunotherapy is now being increasingly used in patients with HCC. Of these new cellular immunotherapeutic techniques, the maturation of effector T-cells into TAA-specific killer cells is receiving great attention, especially in conjunction with stimulating them with educated DCs. However, a method for DC preparation and activation of effector T-lymphocytes for killing cancer cells is needed. Accordingly, in order to improve the cytolytic activity of effector T-lymphocytes against HCC, we set forth to activate T-lymphocytes by monocyte-derived DCs pulsed with cell lysate or RNA prepared from HCC cell lines. TAAs prepared from HCC cell lines exhibited excellent antigenicity to activate lymphocytes, as demonstrated by promising tumor killing activity in both the laboratory and in early-phase clinical studies^12,14,15,43^. We, therefore, hypothesized that DCs pulsed with a combination of antigens prepared from different HCC cell lines would activate multiple clones of effector T-lymphocytes to kill heterogeneous cancer cells within tumor mass.

In our setting, we observed slightly different DC phenotypic markers. From the results of other reports in HCC^11^ and colorectal cancer^28^, levels of co-stimulatory molecules on mDCs were marginally increased, but not significantly different in various antigen presentation conditions. Differences in antigen sources and cancer cell types may explain the variable expression levels of these phenotypic markers compared to the results of our investigation.

The significant expansion of CTLs, NK cells, and NKT cells in lymphocyte population was found to be correlated with improvement in cytolytic activity (Fig. 4). Moreover, IFNγ production was significantly increased in the effector CTLs (Fig. 3C,D). All of these cells play a crucial role in cancer cell elimination^25,44–46^; therefore, increases in the numbers of CTLs, NKT, and NK cells together with improvement in IFNγ production all helped to enhance the eradication HCC cells, as shown in the results of our study. An increase in CTLs was observed in previous studies in HCC^11^ and CCA cell lines^29^. The ability to eliminate cancer cells was also associated with increased IFNγ-producing CTLs^11,29^. In this study, T-cells stimulated with PMA and ionomycin resulted in increased IFNγ production. However, CD4^+^ T-cells were decreased when exposed with these stimulators^47^, which were also observed in this study (Fig. 3A). It should be noted that the number of helper T-cells was not significantly increased when compared to the non-activated lymphocytes (Fig. 2A). However, the production of IFNγ was markedly increased in effector helper T-cells (Fig. 3A,B). This suggests that polarization of type 1 helper T-cells (T_h_1) may occur after the activation by DCs. These T_h_1 helper T-cells have been identified as the functional T-lymphocyte population that provides several cytokines that promote the activation, expansion, and persistence of CTLs^25^.

A previous study by our group revealed that CCA cell line lysate contains several immune suppressive cytokines, such as IL10 and TGFβ ^48^. However, RNAs might possess at least two effects. After up-taking into DCs by macropinocytosis^49^, RNAs could bind to Toll-like receptor (TLR)3, TLR7, and TRL8^50^ to activate DC functions. Moreover, mRNAs in the pooled RNAs could also be translated into intracellular proteins, which are further processed and presented by MHC class I to activated CD8 T-cells^49^. This may explain our finding that effector T-lymphocytes after activation with DCs pulsed by total RNA that was prepared from a single cell line exhibited more cytotoxic activity than that of the total cell lysates in all three HCC target cells (Fig. 4). This finding is consistent with the findings reported from previous studies that used HCC^11^ and CCA cell lines^29^.

Multiple antigen sources from two or three different HCC cell lines were used to broaden activation and enhance the cytotoxic competency of effector T-lymphocytes. Our results showed that the cytotoxic activities of effector immune cells on all HCC target cell lines were correlated with the additional antigen sources used to pulse DCs (Fig. 4). The greatest cytotoxic activity, which increased two-fold compared to antigen from single cell line, was observed in the combination of TAAs prepared from three cell lines (Fig. 4). It should be noted that Huh7 cell line showed the highest cytotoxicity when it was compared with other two HCC cell lines, HepG2 and SNU449 (Fig. 4). Huh7 cell has an epithelial feature and is defined as a well differentiated HCC. Kim M., *et al*. demonstrated that among three HCC cell lines (Huh7, HepG, and SNU449), Huh7 cells showed highest apoptotic induction by NK cells after sensitization with anisomycin^51^. Moreover, MHC Class I chain-related A (MICA) and MICB which are NK cell recognition ligands are highly expressed on Huh7 cell surface^52^. Together with our result that showed the highest apoptotic induction of Huh7 cells (Fig. 4), it may imply that Huh7 cells are more sensitive for cytolytic induction by immune cells than others HCC cell lines. The result of apoptotic activity of HCC cell lines induced by effector T-cells were consistent in all 5 healthy donors (Fig. S5). This is consistent with a study in CCA cell line by Junking *et al*., that found that pooled messenger RNA prepared from three different cell lines promoted the greatest cytotoxic activity by effector T-lymphocytes. In addition, different combinations of antigen sources from CCA cell lines produced distinctive cytotoxic abilities of immune cells^29^. Different cancer cell types may lead to different effects on the cytotoxic activities of effector T-cells after activation by DCs pulsed with different combinations of TAA sources (Fig. 4). Our study demonstrated *in vitro* cytotoxic T-lymphocyte activation by RNA-pulsed DCs, which was partly demonstrated by increased IFNγ levels (Fig. 3). Although the killing mechanism of cytotoxic T-lymphocytes against HCC cells was not investigated, the generally accepted mechanism is their expression and regulated secretion of potent toxins, including pore-forming protein perforin and serine protease granzymes after formation of immunological synapse from the binding of T-cell receptor (TCR) to peptide ligand presented on MHC molecule on the cancer cells. Further *in vivo* study is required to investigate the efficacy of effector T-lymphocytes activated by RNA-pulsed DCs for cellular immunotherapy of HCC.

Some limitations exist in the present study. Firstly, non-tumorigenic HCC cell line, which will serve as negative control for specific killing was not included in the experiments. Secondly, when using tumor RNA or lysate, epitopes of TAAs presented by DCs are unidentifiable. Therefore, not only the efficacy of this treatment strategy, but also its safety requires further investigation. Thirdly, the use of recombinant cytokines is time-consuming, complicated, and expensive, and this is an important concern when DC-based therapy is employed. However, Sundarasetty *et al*. recently reported the use of self-derived DCs for melanoma treatment, in which polyprotein genes encoding GM-CSF, IL4, and TRP2 were cloned into lentiviral vector for transduction into monocytes to generate “Smart-DCs”. The transduced monocytes were self-differentiated into mDCs and simultaneously presented TRP2 antigenic epitopes to T-lymphocytes^53^. Thus, supplementation of recombinant cytokines and exogenous antigen pulsing are not required. Moreover, the time needed for culturing DCs is also reduced. The efficiency of Smart-DCs relative to cytotoxic T-cell activation and killing ability in HCC requires further study. Our group have also successfully developed self-differentiated monocyte-derived dendritic cells (SD-DC) presenting a tumor associated antigen - PRKAR1A (PR), which is an overexpressed in CCA. DCs transduced with lentivirus harboring SD-DC-PR could produce GM-CSF, IL-4 and PRKAR1A. Autologous effector T cells (CD3^+^, CD8^+^) activated by SD-DC-PR exhibited greater cytotoxic activity against CCA than those activated by conventionally-derived DCs^54^.

This study provides a proof-of-concept illustrating that effector T-lymphocytes can be generated by *in vitro* activation of DCs pulsed with pooled RNAs, which serve as the source of TAAs. To further improve this therapeutic approach for HCC, more specific effector T-lymphocytes can be generated by *in vitro* activation with monocyte-derived DCs pulsed with pooled peptides obtained from neo-antigens encoded by mutated genes associated with HCC identified in individual patient. The information of neo-antigens and specific peptides that can be presented by MHC molecule of an individual patient can be predicted from the mutated genes obtained from genomic or transcriptomic sequence of the patient’s cancer cells compared with that of normal cells or identified from whole peptide ligands (ligandome) binding to MHC molecules. These effector T-lymphocytes specific to neo-antigens of HCC will be more effective to target and eliminate cancer cells that can be used singly or in combination with anti-immune checkpoint antibodies or other anti-cancer drugs.

In conclusion, DCs pulsed with multiple sources of antigens improve the cytotoxic activities of effector T-lymphocytes to kill different HCC cell lines, which suggests that this approach be further developed and applied for adoptive T-cell transfer treatment of HCC.

## Materials and Methods

### Ethical consideration

The research proposal and all study protocols were approved by the Siriraj Institutional Review Board (SIRB) of the Faculty of Medicine Siriraj Hospital, Mahidol University, Bangkok, Thailand (COA no. *Si* 414/2015). All the methods in the present study were performed in accordance with the approved guidelines. All healthy volunteers provided written informed consent prior to blood sample collection.

### Cell culture

HepG2 and SNU449 cells were purchased from American Type Culture Collection (ATCC) (Manassas, VA, USA), and Huh7 cells were obtained from Japanese Collection of Research Bioresources (JCRB) (Ibaraki City, Osaka, Japan). HepG2 and Huh7 cells were cultured in complete Dulbecco’s Modified Eagle’s Medium (DMEM) (Gibco; Thermo Fisher Scientific, Waltham, MA, USA) supplemented with 10% fetal bovine serum (FBS) (Gibco) and 50 units penicillin/50 µg streptomycin (Thermo Fisher Scientific). SNU449 cells were cultured in Roswell Park Memorial Institute (RPMI) 1640 Medium (Gibco) supplemented with 10% FBS and 50 units penicillin/ 50ug streptomycin. Cells were placed in a 37 °C incubator saturated with 5% CO_2_ and 60% relative humidity (RH). Standard trypsinization procedure was used to harvest cultured cells.

### Preparation of monocyte-derived dendritic cells

Fifty ml of whole blood was collected from each of 5 healthy volunteers. Peripheral blood mononuclear cells (PBMCs) were prepared by overlaying the whole blood on Lympho-phep (Alere Technologies GmbH, Jena, Germany) and centrifuging at 800 g for 30 minutes without acceleration, deceleration, or stopping. The intermediate layer of PBMCs was collected and then washed with phosphate buffer saline (PBS). PBMCs were then harvested by centrifugation at 380 g for 10 min, and red blood cell contamination was removed by adding red cell lysis buffer. PBMCs were finally washed with serum-free RPMI 1640. AIM-V medium (Gibco) was added to re-suspend PBMCs. The number of PBMCs was counted by trypan blue exclusion assay. PBMCs were placed into a 100 mm culture dish and monocytes were allowed to attach to the surface of the culture container for 2 hours in a 37 °C, 5% CO_2_, and 60% RH environment. Non-adhering lymphocytes were then collected and resuspended in 10% dimethyl sulfoxide (DMSO) in human AB serum (Sigma-Aldrich Corporation, St. Louis, MO, USA) and cryopreserved in liquid nitrogen. Adhering cells, which were mostly monocytes, were subsequently transduced to immature DCs (iDCs) by culturing in AIM-V medium supplemented with 50 ng/ml of GM-CSF (Invitrogen, Carlsbad, CA, USA) and 20 ng/ml of IL4 (Immunotools, Friesoythe, Germany). The fresh culture media was changed every 2 days until day sixth. Then, iDCs were pulsed with TAAs, including total lysate, or RNAs prepared from a single cell line, or combinations of two or three HCC cell lines. The concentration of TAAs from either one cell line or combination of two or three cell lines was 250 µg for total lysate and 20 µg for total RNAs. iDCs were activated to become mature DCs (mDCs) by culturing in AIM-V media supplemented with 20 ng/ml of TNFα and 20 ng/ml of IFNγ (both from Immunotools) for 2 days.

### Activation of effector lymphocytes

Autologous lymphocytes were thawed and the freezing media was immediately removed. The autologous lymphocytes were then co-cultured with mDCs (lymphocyte:DC ratio 10:1) in AIM-V medium supplemented with 5% human AB serum for 2 days. The activated lymphocytes were maintained in AIM-V medium containing 50 units penicillin and 50 µg streptomycin, supplemented with 5% human AB serum, 20 ng/ml of IL2, 10 ng/ml of IL7, and 20 ng/ml of IL15 (all cytokines from Immunotools). Fresh culture media was changed every 2 days until day 10, after which effector T-lymphocytes were ready for further experiments. All cells were cultured in a 37 °C, 5% CO_2_, and 60% RH environment.

### Cytotoxicity assay

Target cancer cell lines were plated overnight. Effector T-lymphocytes were counted and co-cultured with target cells at three different ratios (effector:target 2.5:1, 5:1, and 10:1) for 24 hours. All cells were collected and stained with AnexinV-APC/PI according to the manufacturer’s recommended procedure (Immunotools). The apoptosis signals (AnexinV-APC^+^ and PI^+^ and AnexinV-APC^+^ and PI^-^) of target cells were measured by flow cytometry. The cytotoxic activities of effector T-lymphocytes were summarized from the percentage of target cells that were driven to apoptosis by the effector T-lymphocytes.

### Examination of surface markers by flow cytometry

Monocytes, iDCs, and mDCs were detached from the culture dish using 5 mM EDTA in PBS. Cell surface markers, including CD11c, CD14, CD40 CD83, CD86, and HLA-DR, were examined. Antibodies conjugated with fluorochromes for staining of cell surface markers, including anti-CD11c-APC (Clone BU15), anti-CD14-FITC (Clone 18D11), anti-CD40-FITC (Clone HI40a), and anti-HLA-DR-FITC (Clone MEM12), were purchased from Immunotools. Anti-CD83-PE (Clone HB15e) and anti-CD86-PE (Clone IT2.2) were purchased from eBioscience (San Diego, CA, USA).

Lymphocyte surface markers, including CD3, CD4, CD8, and CD56, were also examined. Antibodies conjugated with fluorochromes for staining of cell surface markers, including anti-CD3-FITC (Clone UCHT-1), anti-CD4-APC (Clone MEM241), and anti-CD8-APC (Clone MEM31), were purchased from Immunotools, and anti-CD56-PE (Clone 5.1H11) was purchased from eBioscience.

Isotype antibodies, including IgG1-PE (Clone PPV06), IgG1-FITC (Clone 203, IgG1-APC (Clone PPV06), and IgG2a-APC (Clone X5563), were purchased from Immunotools for use as staining controls.

Cells were stained with antibodies for 30 min, after which excess antibodies were removed by washing twice with 2% FBS in PBS. All stained cells were immediately analyzed by BD FACSCallibure^TM^ (BD Biosciences, Franklin Lakes, NJ USA) for the acquisition of fluorescent signals. Raw data were annotated and analyzed by FlowJo program version 10.0 (FlowJo LLC, Ashland, OR, USA).

### Examination of intracellular IFNγ by flow cytometry

Fifty-thousand lymphocytes were activated with 50 ng/ul of PMA (Sigma-Aldrich) and 1 µg/ml of ionomycin (Sigma-Aldrich) for 12 hours. A 1x concentration of monensin (eBioscience) was then added to the culture media and the cells were incubated for an additional 12 hours. Activated lymphocytes were collected and stained with anti-CD3-FITC, anti-CD4-APC, and anti-CD8-APC, and the lymphocyte sub-populations were gated by BD FACSCallibure^TM^ (BD Biosciences). After washing, cells were fixed with 1% paraformadehyde in PBS on ice for 15 min. Stained cells were permeabilized with 0.5% saponin and stained with anti-IFNγ-PE (Clone B27; Immunotools) for 30 min, after which the cells were washed and fixed. The fluorescent signals were acquired by flow cytometry and analyzed by FlowJo program version 10.0.

### Statistical analysis

Mean ± standard error of the mean (SEM) of data collected from three independent experiments was calculated. All raw data were annotated and analyzed by GraphPad Prism program version 7.0 (GraphPad Software, Inc., San Diego, CA, USA). Statistical analyses were performed using one-way analysis of variance (ANOVA) with Tukey’s correction for statistical hypothesis testing in multiple comparisons. A *p*-value less than 0.05 indicated significant rejection of the null hypothesis.

## Supplementary information

Supplementary Figure
