# Supplementary material for: Cytolytic Activity of Effector T-lymphocytes Against Hepatocellular Carcinoma is Improved by Dendritic Cells Pulsed with Pooled Tumor Antigens
Source: Sci Rep. 2019 Nov 27;9:17668. doi: 10.1038/s41598-019-54087-0 (PMC6881468; doi:10.1038/s41598-019-54087-0)
Supplement: Supplementary file 1 — Supplementary Figure [file 41598_2019_54087_MOESM1_ESM.pdf]

Cytolytic Activity of Effector T-lymphocytes Against Hepatocellular Carcinoma is Improved by Dendritic Cells Pulsed with Pooled Tumor Antigens

Thaweesak Chieochansin[1], Chutamas Thepmalee[1,2,3], Janya Grainok[1,4], Mutita Junking[1], Pa-thai Yenchitsomanus[1*]

[1] Siriraj Center of Research Excellence for Cancer Immunotherapy, Faculty of Medicine Siriraj Hospital, Mahidol University, Bangkok, Thailand

[2] Graduate Program in Immunology, Department of Immunology, Faculty of Medicine Siriraj Hospital, Mahidol University, Bangkok, Thailand

[3] Division of Biochemistry, School of Medical Science, University of Phayao, Phayao, Thailand.

[4] International Graduate Program in Medical Biochemistry and Molecular Biology, Department of Biochemistry, Faculty of Medicine Siriraj Hospital, Mahidol University, Bangkok, Thailand

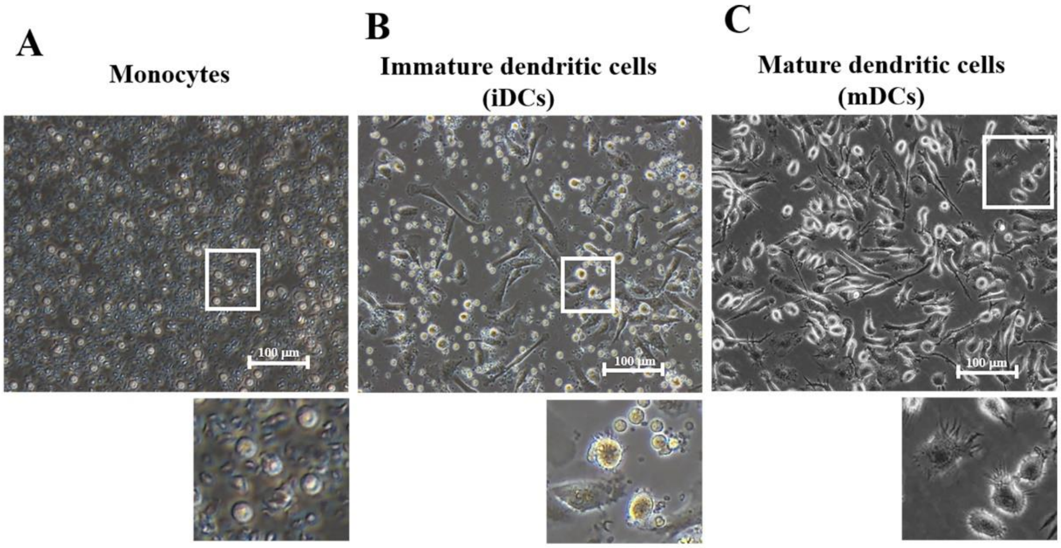

Supplementary Figure S1. Morphology of monocyte-derived dendritic cells. Adherent peripheral blood mononuclear cells (PBMCs) were transduced to become immature dendritic cells (iDCs) by cultivation in AIM-V medium supplemented with GM-CSF and IL4 for 6 days. iDCs were then further differentiated into mature dendritic cells by culturing in AIM-V medium supplemented with TNFα and IFNγ in the presence of antigen for 2 days. Cell morphologies of monocytes (A), iDCs (B), and mDCs (C) were observed under light microscope (10x magnification).

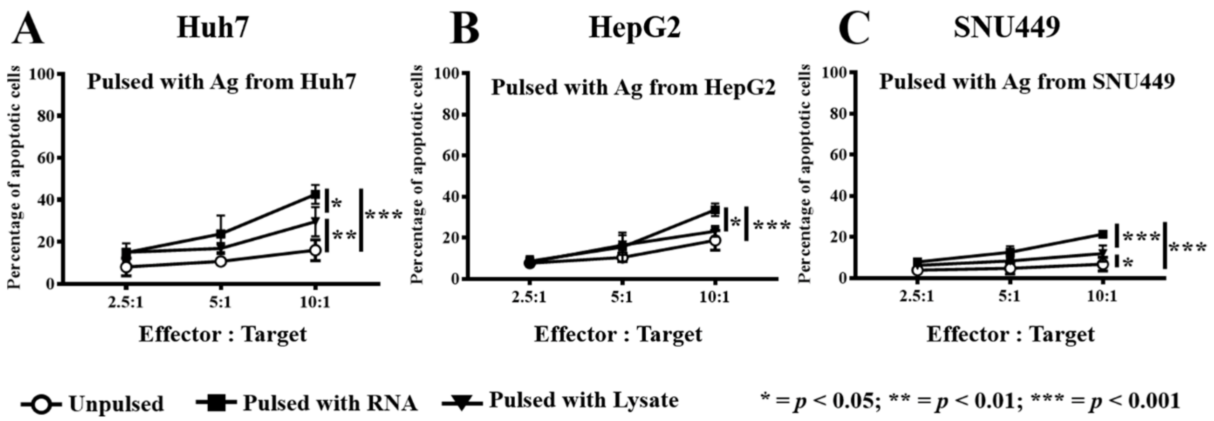

Supplementary Figure S2 Cytotoxic activity of effector T-lymphocytes against HCC cell lines, including Huh7, HepG2, and SNU449. HCC cell line (target cells: T) was co-cultured with effector lymphocytes (effector cells: E) activated by DCs that were pulsed with total RNA or total cell lysate prepared from Huh7 cells (A), HepG2 cells (B), or SNU449 cells (C) at E:T ratios of 2.5:1, 5:1, and 10:1. After 24 hours of co-cultivation, cells were collected and stained with annexin V and PI to determine apoptotic cells by flow cytometry. Data show percentages (mean ± SEM) of target cell apoptosis that were calculated from three independent experiments. (*$p<0.05$, **$p<0.01$, ***$p<0.001$ analyzed by one-way ANOVA with Tukey's correction).

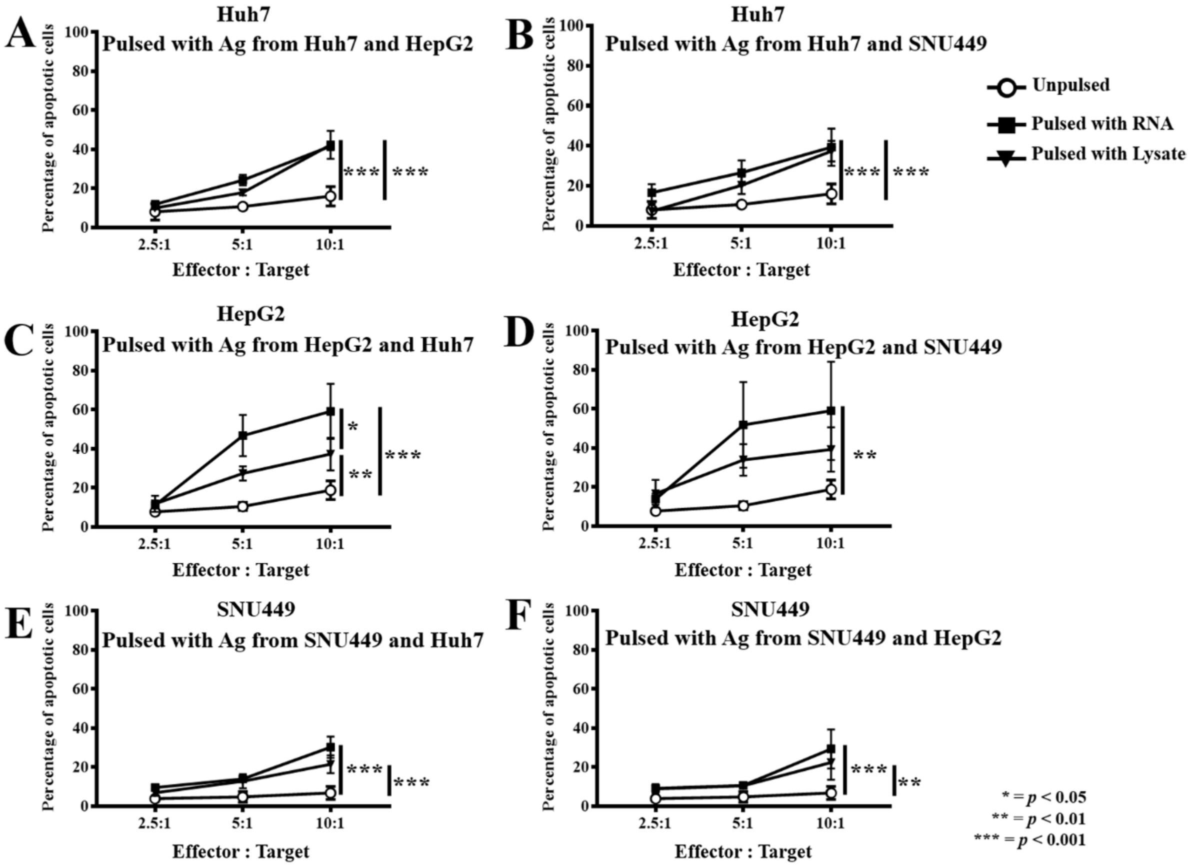

Supplementary Figure S3 Cytotoxic activity of effector T-lymphocytes against HCC cell lines, including Huh7, HepG2, and SNU449. HCC cell line (target cells: T) was co-cultured with effector lymphocytes (effector cells: E) activated by DCs that were pulsed with total RNA or total cell lysate prepared from the combination of two different cell lines. Using Huh7 as the target cell type, the combinations of antigen sources prepared from Huh7 and HepG2, and Huh7 and SNU449 are indicated as (A) and (B), respectively. When HepG2 was the target cell type, the combinations of antigen sources prepared from HepG2 and Huh7, and HepG2 and SNU449 are indicated as (C) and (D), respectively. The combinations of antigen sources prepared from SNU449 and Huh7, and SNU449 and HepG2 are shown as (E) and (F), respectively. Data shows percentages (mean ± SEM) of target cell apoptosis by annexin V and PI staining, and then by flow cytometry. The apoptosis profiles were calculated from three independent experiments. (*$p<0.05$, **$p<0.01$, ***$p<0.001$ analyzed by one-way ANOVA with Tukey's correction).

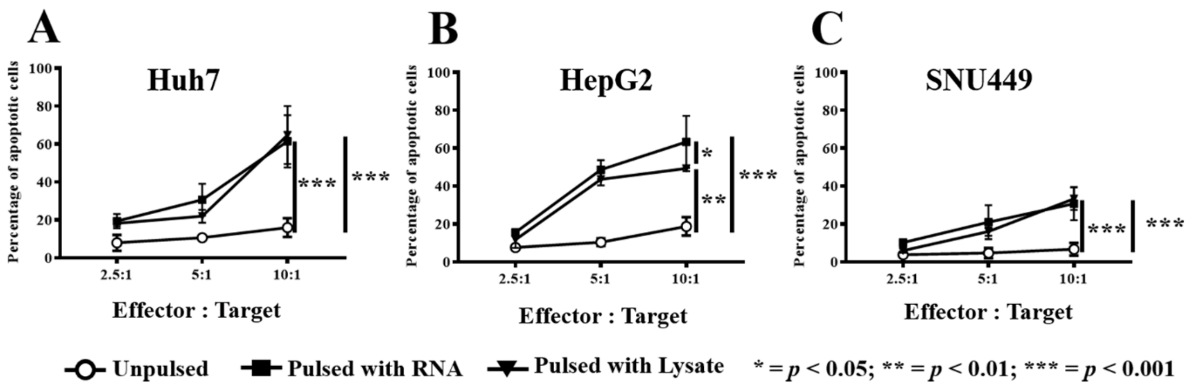

Supplementary Figure S4 Cytotoxic activity of effector T-lymphocytes against HCC cell lines, including Huh7, HepG2, and SNU449. HCC cell line (target cells: T) was co-cultured with effector lymphocytes (effector cells: E) activated by DCs pulsed with total RNA or total cell lysate prepared from the combination of three difference cell lines. After 24 hours of co-cultivation, target cells, including Huh7 (A), HepG2 (B), and SNU449 (C), were collected and stained with annexin V and PI to determine apoptotic cells by using flow cytometry. Data shows percentages (mean ± SEM) of target cell apoptosis that were calculated from three independent experiments. (**p<0.05, **p<0.01, ***p<0.001* analyzed by one-way ANOVA with Tukey's correction).

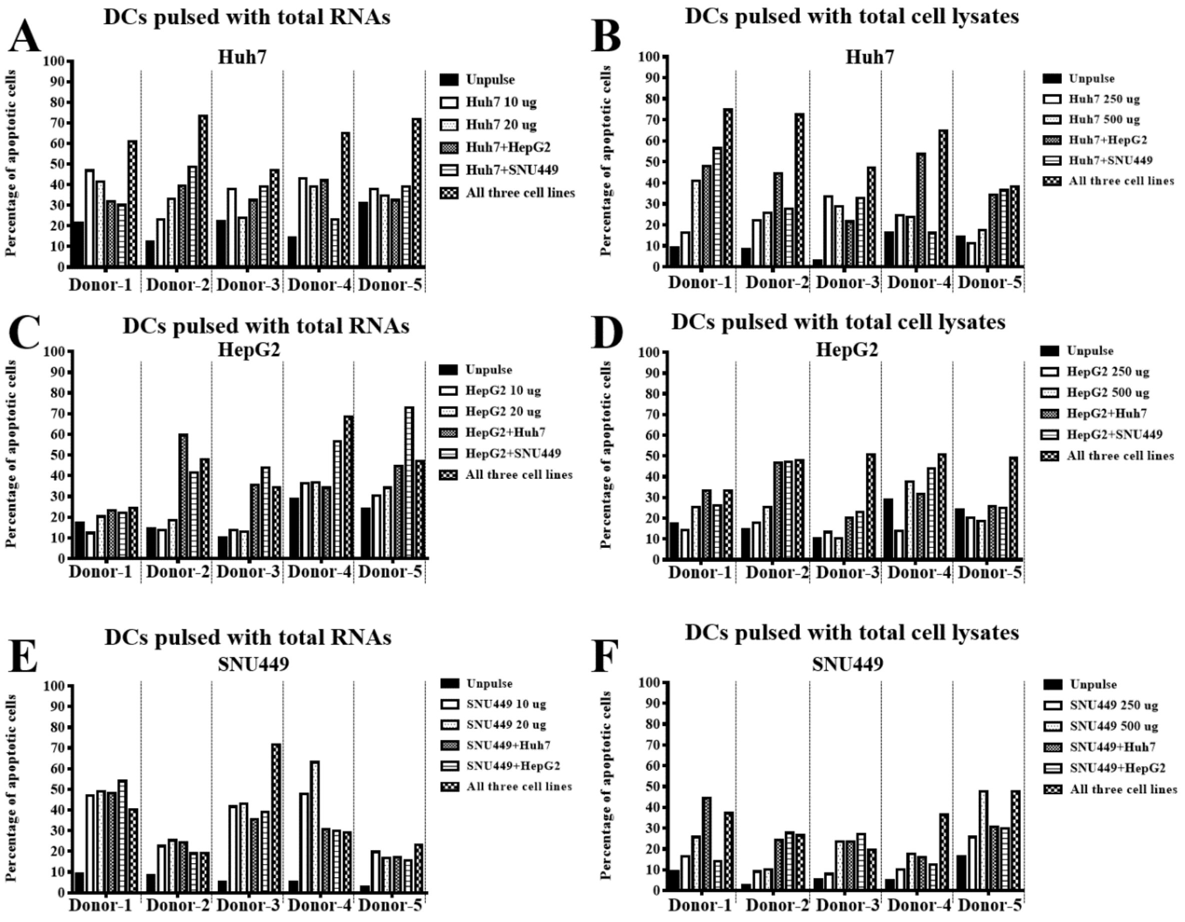

Supplementary Figure S5 Cytotoxic activity of effector T-lymphocytes against HCC cell lines, including Huh7, HepG2, and SNU449. HCC cell line (target cells: T) was co-cultured with effector lymphocytes (effector cells: E) activated by DCs pulsed with total RNA or total cell lysate prepared from the combination of three difference cell lines. The E:T ratio was 10:1. After 24 hours of co-cultivation, target cells, including Huh7 (A was pulsed with RNA and B was pulsed with lysate), HepG2 (C was pulsed with RNA and D was pulsed with lysate), and SNU449 (E was pulsed with RNA and F was pulsed with lysate), were collected and stained with annexin V and PI to determine apoptotic cells by using flow cytometry.
